# Reduction of Metal Artifact in Single Photon-Counting Computed Tomography by Spectral-Driven Iterative Reconstruction Technique

**DOI:** 10.1371/journal.pone.0124831

**Published:** 2015-05-08

**Authors:** Radin A. Nasirudin, Kai Mei, Petar Panchev, Andreas Fehringer, Franz Pfeiffer, Ernst J. Rummeny, Martin Fiebich, Peter B. Noël

**Affiliations:** 1 Department of Diagnostic and Interventional Radiology, Technische Universität München, Ismaninger Strasse 22, 81675 Munich, Germany; 2 Institut für Medizinische Physik und Strahlenschutz, Technische Hochschule Mittelhessen, Wiessenstrasse 14, 35390 Giessen, Germany; 3 Chair for Biomedical Physics and Institute for Medical Engineering, Technische Universität München, James-Franck-Strasse 1, 85748 Garching, Germany; Institute of Automation, Chinese Academy of Sciences, CHINA

## Abstract

**Purpose:**

The exciting prospect of Spectral CT (SCT) using photon-counting detectors (PCD) will lead to new techniques in computed tomography (CT) that take advantage of the additional spectral information provided. We introduce a method to reduce metal artifact in X-ray tomography by incorporating knowledge obtained from SCT into a statistical iterative reconstruction scheme. We call our method Spectral-driven Iterative Reconstruction (SPIR).

**Method:**

The proposed algorithm consists of two main components: material decomposition and penalized maximum likelihood iterative reconstruction. In this study, the spectral data acquisitions with an energy-resolving PCD were simulated using a Monte-Carlo simulator based on EGSnrc C++ class library. A jaw phantom with a dental implant made of gold was used as an object in this study. A total of three dental implant shapes were simulated separately to test the influence of prior knowledge on the overall performance of the algorithm. The generated projection data was first decomposed into three basis functions: photoelectric absorption, Compton scattering and attenuation of gold. A pseudo-monochromatic sinogram was calculated and used as input in the reconstruction, while the spatial information of the gold implant was used as a prior. The results from the algorithm were assessed and benchmarked with state-of-the-art reconstruction methods.

**Results:**

Decomposition results illustrate that gold implant of any shape can be distinguished from other components of the phantom. Additionally, the result from the penalized maximum likelihood iterative reconstruction shows that artifacts are significantly reduced in SPIR reconstructed slices in comparison to other known techniques, while at the same time details around the implant are preserved. Quantitatively, the SPIR algorithm best reflects the true attenuation value in comparison to other algorithms.

**Conclusion:**

It is demonstrated that the combination of the additional information from Spectral CT and statistical reconstruction can significantly improve image quality, especially streaking artifacts caused by the presence of materials with high atomic numbers.

## Introduction

Artifacts occur frequently in X-ray Computed Tomography (CT). They are generally described as the discrepancy between the CT numbers of the reconstructed image and the true attenuation coefficients of the object [1]. Artifacts degrade the diagnostic quality of CT images and may result in incorrect evaluation of clinical images. Beam hardening is one of the main sources of artifact in CT, due to the polychromatic nature of x-rays [2, 3]. As the photons penetrate through an object, more low energy photons are absorbed, resulting in a shift of the x-ray spectrum to a higher energy range. This causes two types of artifacts: cupping artifacts and dark streaking between dense objects [1, 3].

One other cause of artifacts is the presence of high Z-materials in the field-of-view such as hip prostheses, metal implants and dental fillings [1, 4]. These materials have high attenuating properties, resulting in the photon starvation phenomenon, whereby the amount of photons reaching the detector is highly reduced [5]. The ‘missing information’ on the projection data cause incorrect calculations during the conventional analytical image reconstruction process, thus leading to severe streaking and dark and bright shading around the metal implant. Various methods have been developed for metal artifact reduction (MAR) [6–12]. One popular technique is in-painting. In this method, regions associated with metal are ‘replaced’ by interpolating neighboring values in the sinogram. [11–13]. In detail, the technique involves masking or segmenting the CT images to obtain the metal only image, forward projecting it, removing the metal-only data from the original sonogram and interpolate the missing sinogram from its neighbors before re-reconstructing the modified sinogram. While this method removes most of the streaking artifacts in the image, it has limited effectiveness, as it is prone to reintroduce other artifacts due to interpolation errors, causing a loss of details especially around metal implants. Another advanced method is to recover the high frequencies of an uncorrected image, which contain edge information and noise, and recombine it with the MAR-corrected image [14]. This technique has been shown to preserve details around the metal implant; however, it is very dependent on the optimal segmentation of the metal from other high frequency components in the image such as bone.

A unique approach from Stayman et al. [5] uses a model-based penalized maximum-likelihood estimation to reduce the effects of metal artifacts. In the method, known as Known Components Reconstruction (KCR), information on the shape and composition of the metal in the scanned object is derived from a computed-assisted design (CAD) model and is incorporated into the iterative reconstruction process as prior. CT images reconstructed using this technique have shown significant reduction of streaking artifacts and dark shading caused by the presence of metal, when mono-energetic x-rays are used.

Advancement in detector technology has contributed to the development of energy-resolved photon counting detectors [15–17]. Photon counting detectors have the ability to discriminate incoming photons based on their energies, hence obtaining the spectral information of the object in a single scan at the same tube voltage [18, 19]. In this technique also known as Spectral CT (SCT) imaging, photon-counting detectors split the x-ray spectrum into several predefined energy bins, enabling the acquisition of separate CT data in each energy bin. With prior knowledge of the materials present in the object, it is possible to perform material decomposition on the projection data [18–20]; therefore additional information of the shape and location of the metal in the object can be determined. Although metal decomposition method is also a common technique in dual-energy CT (DECT) [21], SCT has the advantage that the spectral information can be obtained in a single scan and thus issues such as additional dose and cross-scatter contamination in the case of dual-source CT (DSCT) can be avoided [22].

Our work adopts the same concept of incorporating prior knowledge into the reconstruction algorithm. However, instead of using a CAD model, we utilize the additional information obtained from Spectral CT as a priori for our reconstruction. We present a new algorithm to reduce metal artifacts in CT images based on a two-step approach; in step one our algorithm performs material decomposition on the spectral data to determine the shape and the spatial location of the gold, and step two incorporates that information as a prior into a penalized maximum log-likelihood reconstruction algorithm.

This paper is structured as follows: in the Methods section, we introduce our material decomposition technique and the penalized maximum likelihood reconstruction algorithm. In the Results section we present the reconstructions from our algorithm and compare them to conventional FBP and iterative approaches. Further, we investigate the influence of accurateness of the shape and the spatial location towards the performance of our reconstruction algorithm. Finally, in the Discussion section, we discuss the possibility and the challenge of using our technique in a clinical setting.

## Methods

**Fig 1:**
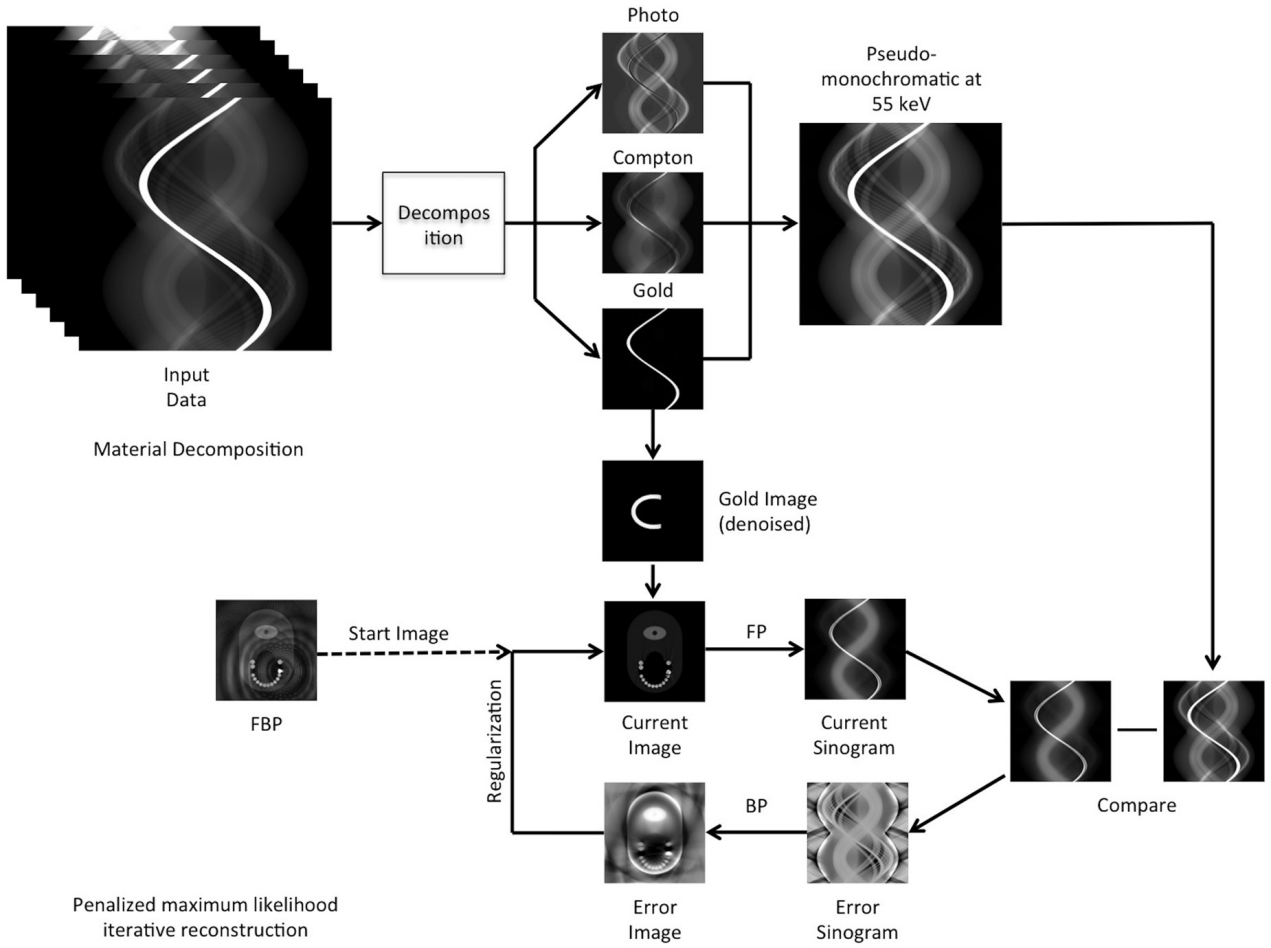
An overview of the SPIR technique. In the first of two steps, the projection data is decomposed into several basis functions from which the pseudo-monochromatic projection is calculated and the metal image is reconstructed. Using these as input and prior respectively, the image is iteratively reconstructed in the second step, while at the same time knowledge on the location and density of the metal implant is enforced and corrected.

An overview of our algorithm is illustrated in Fig 1 as well as in the pseudo code in Algorithm 1. Our algorithm consists of two steps: material decomposition of the Spectral CT data (A) and the penalized maximum likelihood iterative reconstruction (B). We call our algorithm Spectral-driven Iterative Reconstruction (SPIR).

### Material decomposition

The material decomposition method is based on works in [18] and [19].

In diagnostic imaging, x-ray is primarily attenuated by photoelectric absorption and Compton scattering. Photoelectric effect occurs mostly at the lower x-ray energies and can be approximated by the E^-3^ energy dependence [20]. At higher x-ray energies, Compton effect is more dominant and the cross-section can be derived from the Klein-Nishina function [20]. In the presence of materials with distinctive k-edge discontinuity, such as gold, the linear attenuation coefficient can be described as:

$$y(\vec{x},E)=A_{1}(\vec{x})\frac{1}{E^{3}}+A_{2}(\vec{x})f_{KN}(E)+A_{3}(\vec{x})f_{Au}(E)$$ (1)

where *A*_*1*_, *A*_*2*_ and *A*_*3*_ denotes the local density of the basis function, f_KN_ the Klein-Nishina function and f_Au_ the mass attenuation of gold.

In order to estimate the parameters *A*, an equal or more number of x-ray photon counts measurements (i.e. bins) are required. For a photon-counting detector with *N* energy bins, the number of photons *λ*_*n*_ detected in an energy bin can be estimated as:

$$\begin{array}{l} \lambda_{n}(A_{1},A_{2},A_{3})=\int_{0}^{\infty }S_{n}(E)\Phi (E)exp(-A_{1}E^{-3}-A_{2}f_{KN}-A_{3}f_{Au})D(E)dE\\ n=1,.....,N \end{array}$$ (2)

The index *n* refers to the *n* ^*th*^ energy window, while *Φ*(E) denotes the photon flux and *S*_*n*_ *(E)* the spectral response of the detector. *S*_*n*_ *(E)* equals to 1 if the photons are detected in the corresponding bin and 0 if they are elsewhere. In this work, we neglected the effect of detector response *D(E)* because the simulated detector is assumed to be ideal.

As the number of energy bins exceeds the number of basis functions, the system is over-determined. We used the maximum likelihood parameter estimation method to estimate the line integrals of the individual components. Assuming the counting procedure follows a Poisson distribution, the likelihood function given by measurement results (m_1_…m_*N*_) can be written as:

$$P(m_{1}...,m_{n}|\lambda_{1}(A),...,\lambda_{n}(A))=\prod_{n=1}^{N}\frac{\lambda_{n}{\left(A\right)}^{m_{n}}}{m_{n}!}e^{-\lambda_{n}(A)}$$ (3)

With *A*, we mean *A*_*1*_, *A*_*2*_, *A*_*3*_. It is more convenient to minimize the negative log-likelihood [19], therefore we can re-write the likelihood function as a function of parameters *A*, as:

$$\begin{array}{l} L(m_{1}...,m_{n}|\lambda_{1}(A),...,\lambda_{n}(A))=-\text{ln}(P)\\ L(m_{1}...,m_{n}|\lambda_{1}(A),...,\lambda_{n}(A))≅\sum_{n=1}^{N}\left[\lambda_{n}(A)-m_{n}\text{ln}\lambda_{n}(A)\right] \end{array}$$ (4)

This maximum likelihood technique yields the sinograms of photoelectric effect, Compton scattering and the attenuation of gold. From these basis functions, we calculated a pseudo-monochromatic projection data at 55 keV and used as the input of our penalized maximum likelihood iterative reconstruction technique. The calculated pseudo-monochromatic projection data is hereafter notated as y*. At the same time, we reconstructed the gold component of the decomposition using a standard filtered-backprojection (FBP). We performed an image-processing step on the gold image to remove residual noise from the material decomposition process and better localize the position of the metal implant. The image-processing step involves applying zeros on pixels that have values less than 10% of the maximum pixel value of the image. Finally the information on the density and position of the gold implant is passed as a prior into our statistical reconstruction scheme.

### Penalized maximum likelihood iterative reconstruction

For reconstruction we used a penalized maximum likelihood approach. This Poisson-statistics-based algorithm uses the raw measurements rather than the logarithms of the data, and thus it is believed to solve nonlinearity of the logarithm and handle low radiation scans.

The goal of the algorithm is to maximize a cost function ψ, which consists of a likelihood term *L* and regularization term *R*. *L* indicates how the reconstructed result matches the input sinogram;

$$\hat{\mu }=\arg\max\Psi (\mu ),\;\Psi (\mu )=L(\mu )-\beta R(\mu )$$ (5)

where *μ* indicates the image matrix in attenuation values. It describes the probability of how the reconstructed result matches the measurement [23]. R is a regularization term given in [24] and can controlled by β. The regularization term is written as:

$$R(\mu )=\sum_{k}w_{jk}\psi (x_{j}-x_{k})$$ (6)

where *ψ(t)* is the penalty function. The definition of *w*_*jk*_ is given later on.

Adding a penalty term to regularize the problem leads to a faster convergence and an enforcement of desired and beforehand known image properties like smoothness and edge preservation. We used Lange’s [25] function as penalty function *ψ(t)* in the regularization term. Lange’s penalty has the feature to eliminate noise as well as preserve edges in the images.

$$\psi (t)=\delta^{2}\left[\left|t/d\right|-\text{log}(1+\left|t/d\right|)\right]$$ (7)

*δ* is a constant as the edge threshold in denoising.

In order to maximize this optimization problem, we made use of the separable paraboloidal surrogate (SPS) technique [23]. Each update step is given by:

$$\mu^{n+1}={\left[\mu^{n}+\frac{BP\left[bexp(-FP\left[\mu^{*}\right])-y^{*}\right]-\beta \sum_{k}w_{jk}\dot{\psi }(x_{j}-x_{k})}{BP\left[y^{*}FP\left[1\right]\right]+\beta \sum_{k}w_{jk}\ddot{\psi }(x_{j}-x_{k})}\right]}^{+}$$ (8)

where y* is the monochromatic projection data. FP [] and BP [] denote the forward- and backward projection respectively; *w*_*jk*_ *ψ()* the regularization term; *x*_*j*_ are pixels in image *μ*, while *x*_*k*_ are neighboring pixels of *x*_*j*_. *w*_*jk*_ indicates the distance-weight between *x*_*j*_ and *x*_*k*_. []+ denotes the operation of removing negative values. *b* is the x-ray intensity at the source.

Selecting the constants *β* and *δ* for the regularization was done subjectively. The user defined the trade-off point between the data fidelity term and the roughness term. This was done by a specification of a desired target noise level of the reconstructed image.

Prior to each update step as in (8), the location and density of the gold determined in the previous section are pre-computed into exact pixel values in image matrix *μ*, where metal material is located. The modified image matrix with prior information is noted as μ*.

In each update step, a subset of three angles is taken in the forward and back projection to compute an update of *μ*. These angles are randomly chosen from the total number of projections to accelerate the optimization process. In this work, we consider a full iteration when all projections are chosen (360° rotation). Our previous experiments indicate that a convergence can be seen after 10 to 15 full iterations, thus for this work we choose 15 iterations as our stopping criteria.

### Simulation set-up

**Fig 2:**
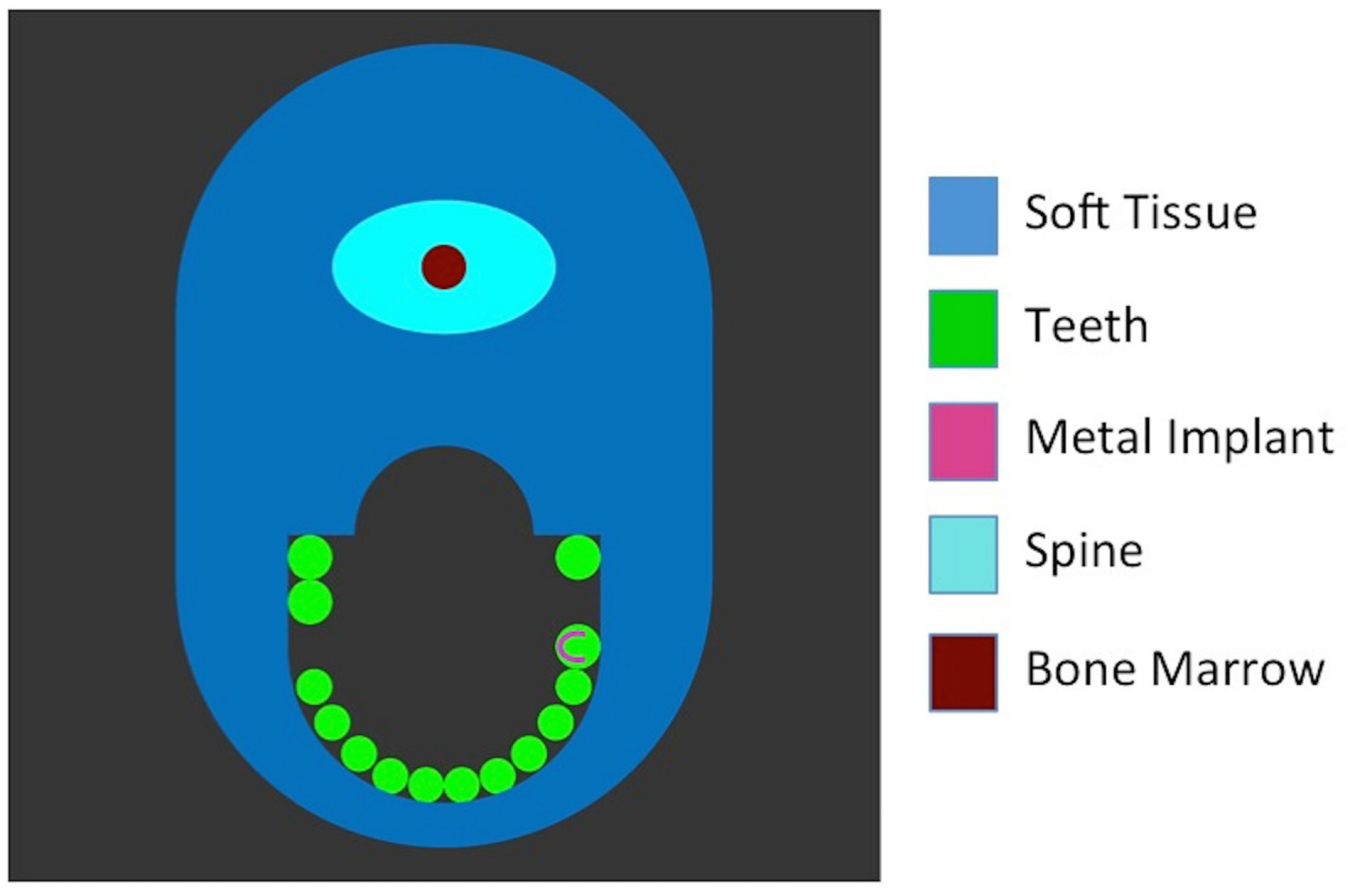
One of the virtual phantoms used in this work. It contains several anatomic components of a jaw such as soft tissue, spine, bone marrow and teeth. A metal implant is embedded on one of the teeth. In this work, we simulated three different shapes of metal implants: circle, horseshoe and triangle.

We simulated a phantom based on the information provided by the Phantom-Group (IMP, University Erlangen-Nürnberg, Erlangen, Germany). It consisted of 16 molars, one of which has a dental implant made of pure gold (density 19.3 g/cm3). Two of the molars were removed to simulate a gap (see Fig 2). In this work, we experimented with different shapes and sizes of dental implants. This was done to test the influence of prior knowledge on the overall performance of our SPIR algorithm. For the simulation, we used a mathematical phantom made with known geometry, instead of a voxel phantom, in order to accelerate the simulation process.

**Fig 3:**
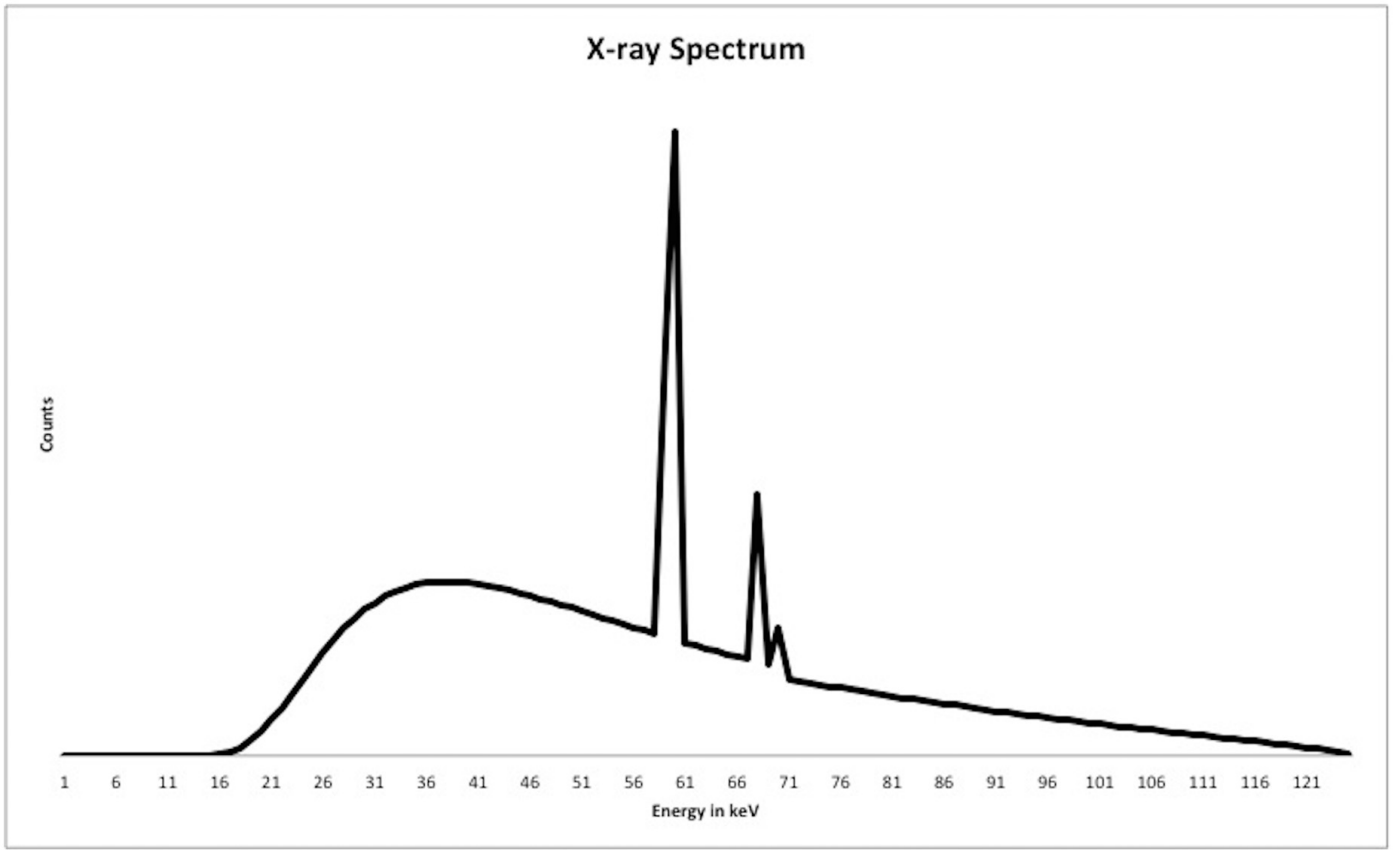
The X-ray spectrum used in the simulation. The x-ray source was generated at tube voltage of 125 kV with Wolfram target and aluminum filter of thickness 2.7mm, yielding mean spectrum energy of 55.457 keV.

The photon transport mechanism was simulated using a Monte-Carlo simulator based on EGSnrc C++ class library [26, 27]. The EGSnrc is a general purpose Monte Carlo package that can be used to simulate the transport of photons and electrons in any arbitrary geometry for particles in the energy range from 1 keV to 10 GeV [27, 28]. In all simulations the Compton interaction was modeled in the impulse approximation [29], the photon cross-section was taken from the XCOM [30] tabulations and all photons were tracked to an energy of 1 keV. The x-ray source was generated at tube voltage of 125 kV with Wolfram target and aluminum filter of thickness 2.7mm, yielding mean spectrum energy of 55.457 keV. A diagram of the spectrum is shown in Fig 3. A total of 3.10x10^10^ photons were released at the source. A clinical dental CT system with a source to detector distance of 80 cm, source to isocenter distance of 60 cm and a detector with 2200x2200 pixels of 0.01 x 0.01 cm^2^ size was modeled. The detector was operated in photon-counting mode with each photon discriminated according to its kinetic energy into 62 bins, with each bin having a bin width of 2 keV. These individual energy bins were summed together after the complete simulation to obtain spectral CT projection data with six energy windows. The implementation of 2 keV energy windows allowed us to choose the optimal number and size of the energy windows after the simulation. This step enabled a significant reduction in computational time. In order to make the simulations as realistic as possible, all physical effects such as Rayleigh scattering, atomic relaxations and photoelectron angular sampling were taken into consideration. The detector was assumed to be ideal, thus effects such as charge sharing and pulse splitting can be neglected. However, we included the two main sources of noise in a photon-counting CT system: quantum noise and noise from scattered photons. Further, one of the advantages of energy-resolved photon-counting detectors is the ability to eliminate electronic noise during acquisition. Thus it can be omitted in our simulation.

### Implementation

Our SPIR algorithm was implemented partly in Matlab (The Mathworks, Inc., Natick MA) with links to in-house developed C++ libraries. For the material decomposition, we used the simplex method (Nelder-Mead simplex direct search method) included in the Optimization Toolbox to solve the negative log-likelihood minimization. Iterative reconstructions are known to be computationally intensive and time-consuming, therefore we implemented the projection and back-projection model as well as the penalty function on a OpenCL platform for execution on a NVIDIA Tesla C1060 graphic card [31, 32]. As a result, we are able to accelerate the reconstruction process and significantly reduce the computation time, while preserving the number of iteration and image quality.

In summary a simplified version of the algorithm can be found as pseudo code in Algorithm 1.

**Algorithm 1** pseudo code

A ← initialize parameters *A_1_*, *A_2_*, *A_3_*

**for** i = 1 to max projection data **do**

**for** j = 1 to number of detector elements **do**

*A* ← $\text{min}\sum_{n=1}^{N}\left[\lambda_{n}(A)-m_{n}\text{ln}\lambda_{n}(A)\right]$ min

**end for**

**end for**

*μ_metal_*← FBP[*A_3_*]

*μ*← initial reconstruction

$d=BP\left[y^{*}\cdot FP\left[1\right]\right]$

**for** k = 0 to max iteration do

**for** m = 1 to number of subset do

$\mu^{*}\leftarrow \mu_{metal}$

$l\leftarrow FP\left[\mu^{*}\right]$

$h\leftarrow b\cdot \exp(-l)-y^{*}$

L←BP[h]

$\mu \leftarrow {\left[\mu +\left(L-\beta \dot{R}\right)/\left(d+\beta \ddot{R}\right)\right]}^{+}$

**end for**

**end for**

## Results

**Fig 4:**
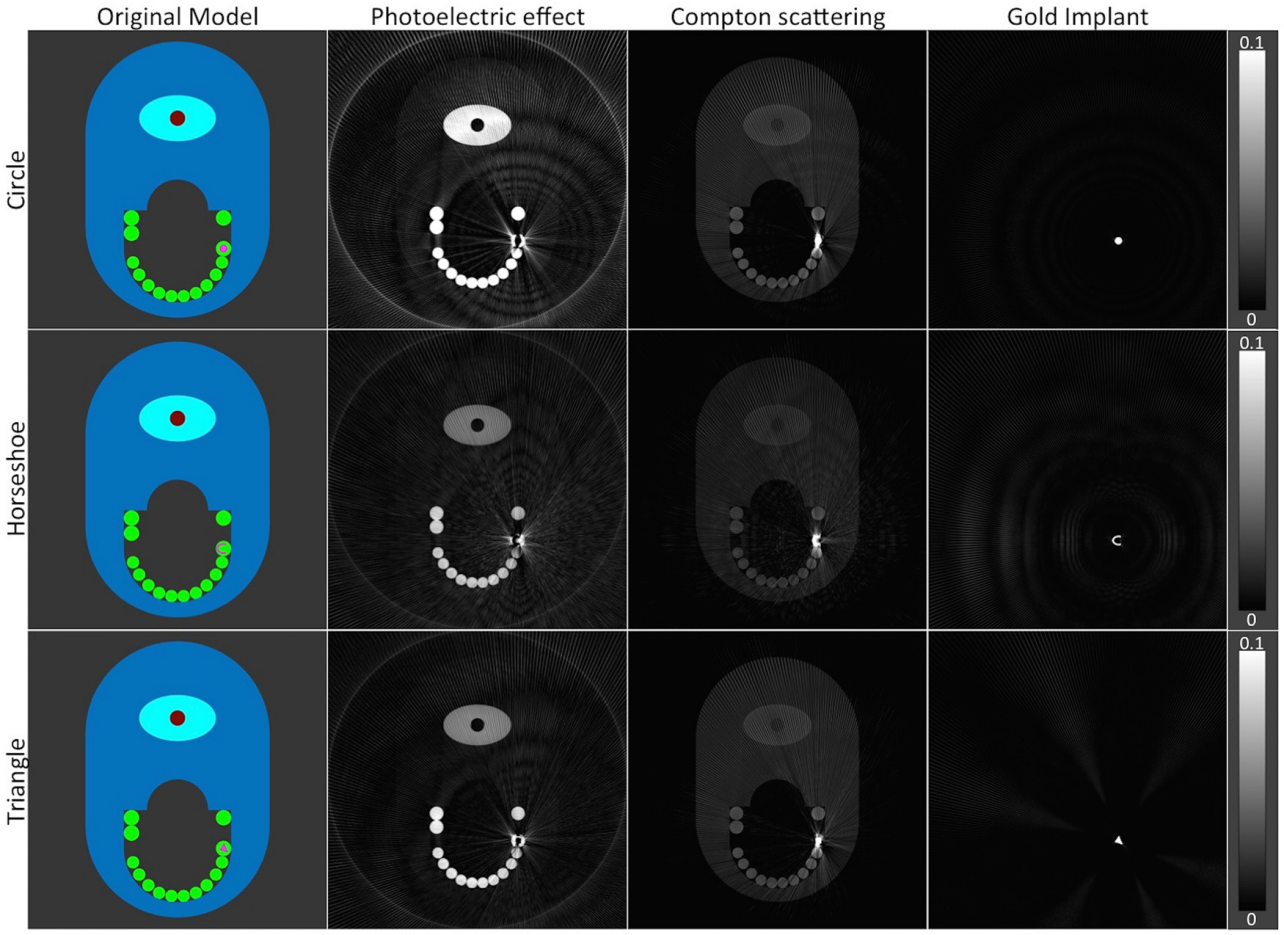
The original model of the phantom and the images of the decomposed basis functions reconstructed with filtered back-projection (FBP). The second column shows photoelectric attenuation, third column the Compton scattering, and the fourth column the gold attenuation. Row-wise are the different shapes of the metal implant: first row circle, second row horseshoe, and bottom row triangle. It can be seen that, the location of the gold implant is accurately detected, while the gold implant can be distinguished from other parts of the phantom, especially the teeth. The reconstructed images are normalized to 1, and have WW of 0.2 and WL of 0.1.

**Fig 5:**
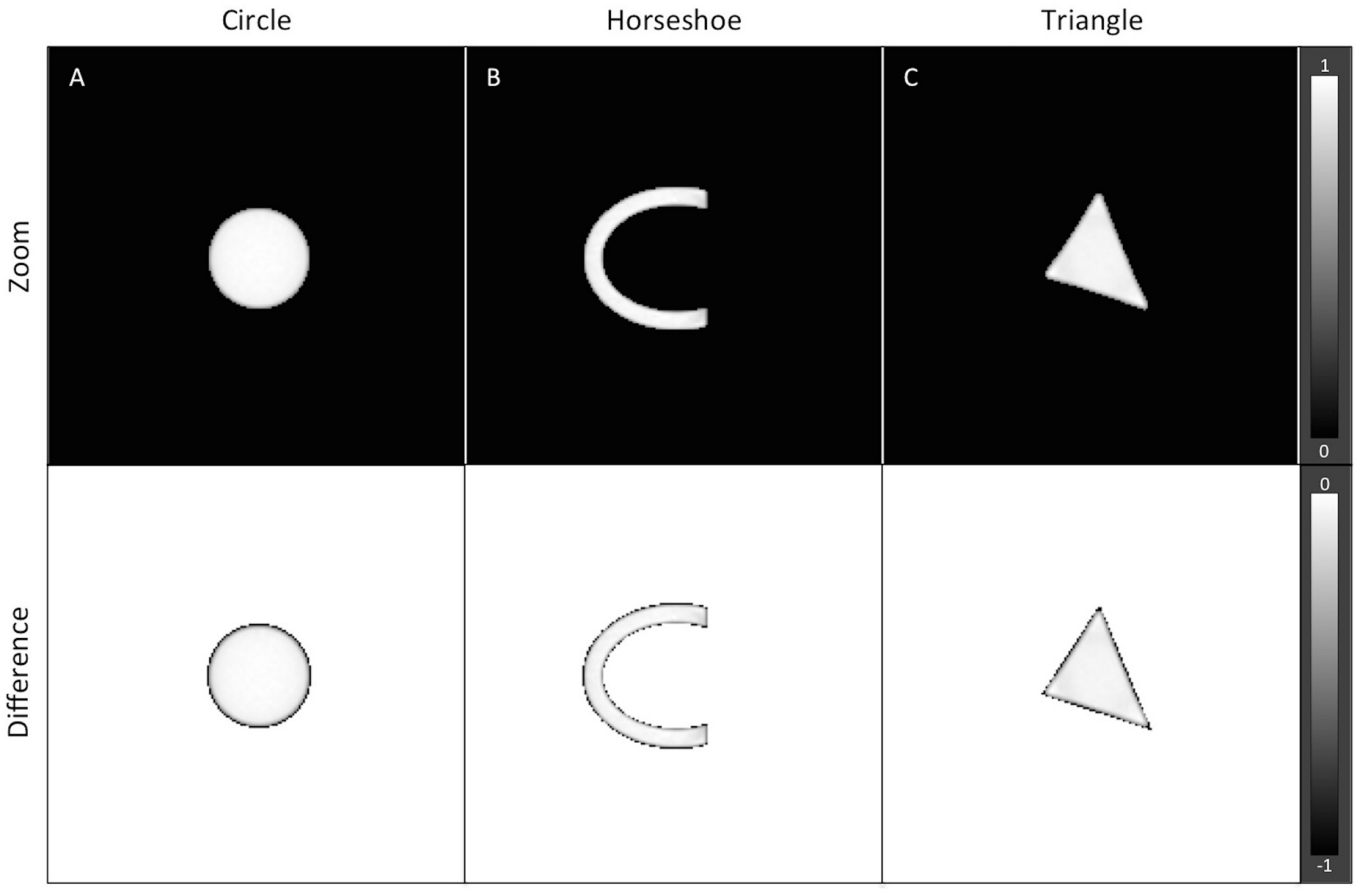
The zoom-in of the metal implants. The zoom-in of the metal implants obtained shown in the top-row indicates the accuracy of the material decomposition technique in detecting the metal implant. When compared to the original model, only a slight difference as in 1 pixel is observed, which can be attributed to discretization error. The average difference in density is about 0.285 g/cm3. The top-row images are normalized to 1, with WW of 1.0 and WL of 0.5. The bottom-row images have WW 1.0 and WL of -0.5.

In the first step of our algorithm, the projection data were first decomposed into three basis functions that describe the total attenuation in the scanned object. Fig 4 illustrates the original model and the results of decomposition into three basis component images: (A) photoelectric effect, (B) Compton scattering and (C) gold attenuation. One can see that the spatial location of the gold implant is accurately detected and distinguished from other anatomic structures of the phantom such as teeth and spine. On a closer visual inspection, the shape of the metal implant is exact and resembles the original model, as illustrated by the top row of Fig 5. Subtracting images of the gold component image from the original model show only a slight difference of 1 pixel between the images, which can be attributed to discretization error in the simulation, indicating the high accuracy of the material decomposition technique. Further, the average difference in density between the decomposed metal and the original model is about 1.5% or 0.285g/cm^3^.

**Fig 6:**
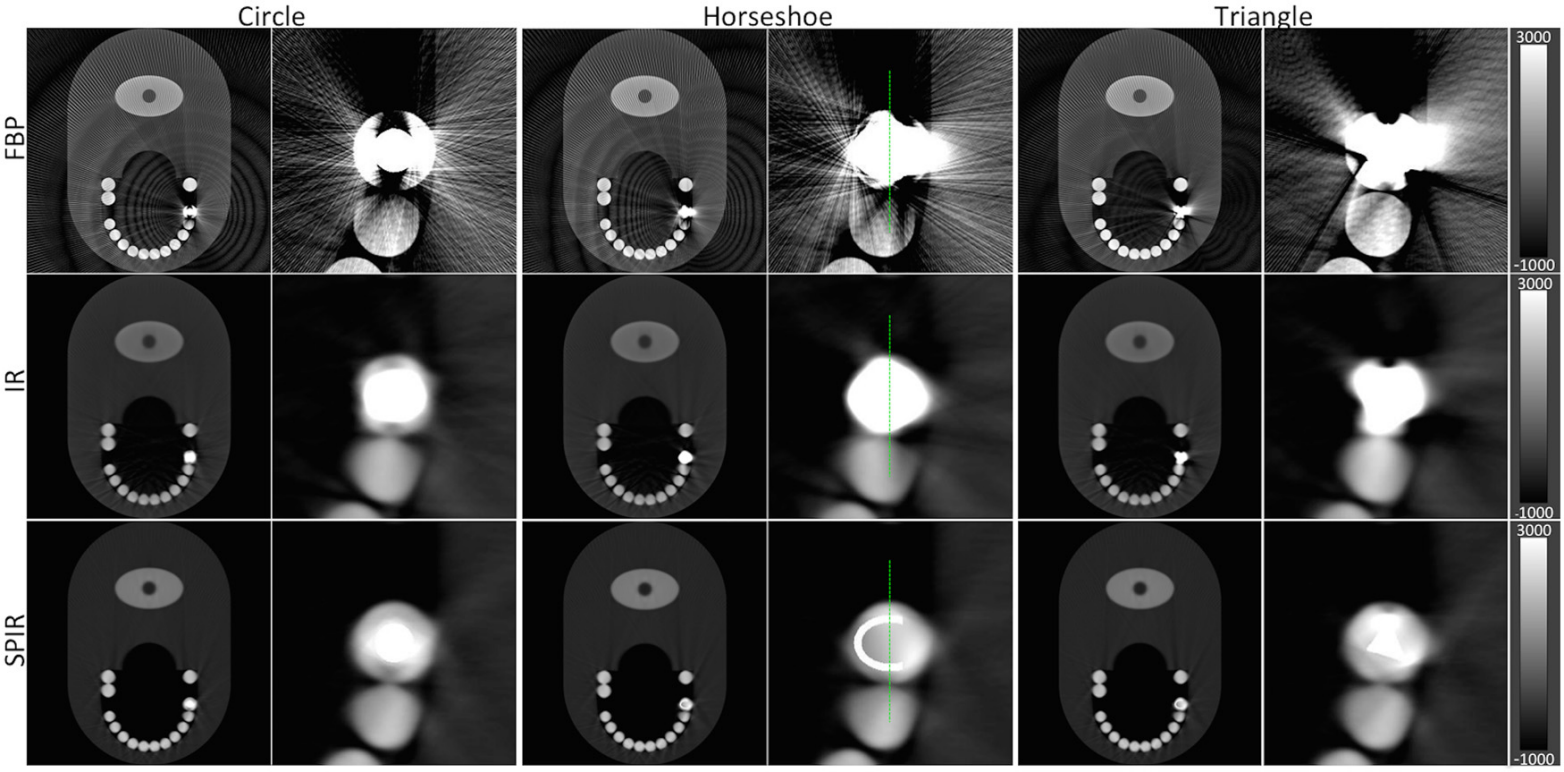
The reconstructions of the phantom using different algorithms. The reconstructions of the first row is done using FBP, second row penalized maximum likelihood iterative reconstruction without prior (IR), and the third row Spectral-driven Iterative Reconstruction (SPIR). Column wise are the different shapes of metal implant at full-view and zoomed-view. The first column-pair has the shape circle, second column-pair horseshoe, and third column-pair triangle. All images have WW of 1000 HU and WL of 4000 HU.

The second step of our algorithm involves the statistical reconstruction of the pseudo-monochromatic data, with the information of the gold component used as a prior. Fig 6 presents the full-view and the zoom-in of the images reconstructed with three different reconstruction algorithms: FBP, penalized maximum likelihood iterative reconstruction on the plain absorption data without prior information (IR), and SPIR. In the first row, the reconstruction using FBP produces images with massive streaks and black and white shadings, especially around the metal implant. The presence of these artifacts severely degrades the diagnostic quality of the image, while at the same time information near the implant is lost. Using the more advanced IR algorithm these artifacts are significantly reduced, as shown by images in the second row; however the dark and bright shadings around the implant are still visible. In the third row one can observe that the incorporation of prior information obtained from the material decomposition technique into the reconstruction algorithm delivers notably improved images: bright streaks are reduced significantly without compromising the anatomical information, while the shadings around the dental implant are considerably eliminated, as displayed in the zoom-in. The SPIR algorithm is not only able to reduce the artifacts, but also preserve the edges and valuable anatomical details near the implant.

**Fig 7:**
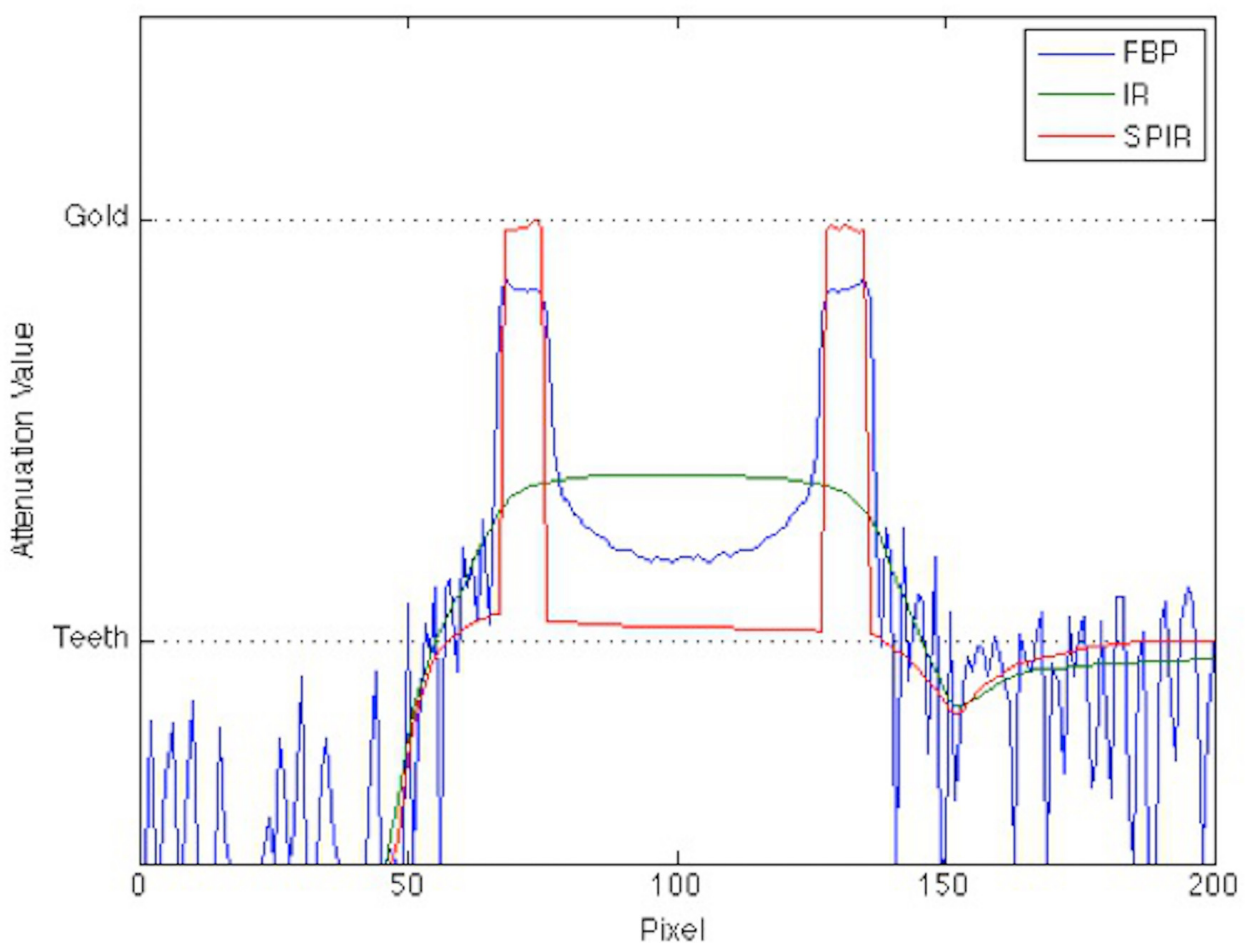
The vertical line profiles as marked in Fig 4 for different reconstruction algorithms. The line profile from SPIR algorithm reflects the true attenuation values best, in comparison to FBP and IR.

We analyze the results quantitatively by selecting a line-of-interest and collected pixel values (attenuation values) along the line, as indicated by the green line in the middle column images of Fig 6, and compare it to the true theoretical values used in the Monte Carlo simulation. Fig 7 illustrates the line profiles of the three reconstruction algorithms. On the y-axis, the theoretical attenuation values of several anatomical components such as teeth and the gold implant are shown as dashed lines. From the graph one can see that the artifacts in the FBP images cause a massive fluctuation of values. The IR algorithm produces a smooth profile but does not return attenuation values similar to the true theoretical one. SPIR algorithm is the only one to produce robustly and reproducibly the true theoretical value.

Computationally, our FBP algorithm needs 5 seconds to reconstruct an image. For the same image the IR and our proposed SPIR algorithm took about 20 minutes for 15 iterations.

**Fig 8:**
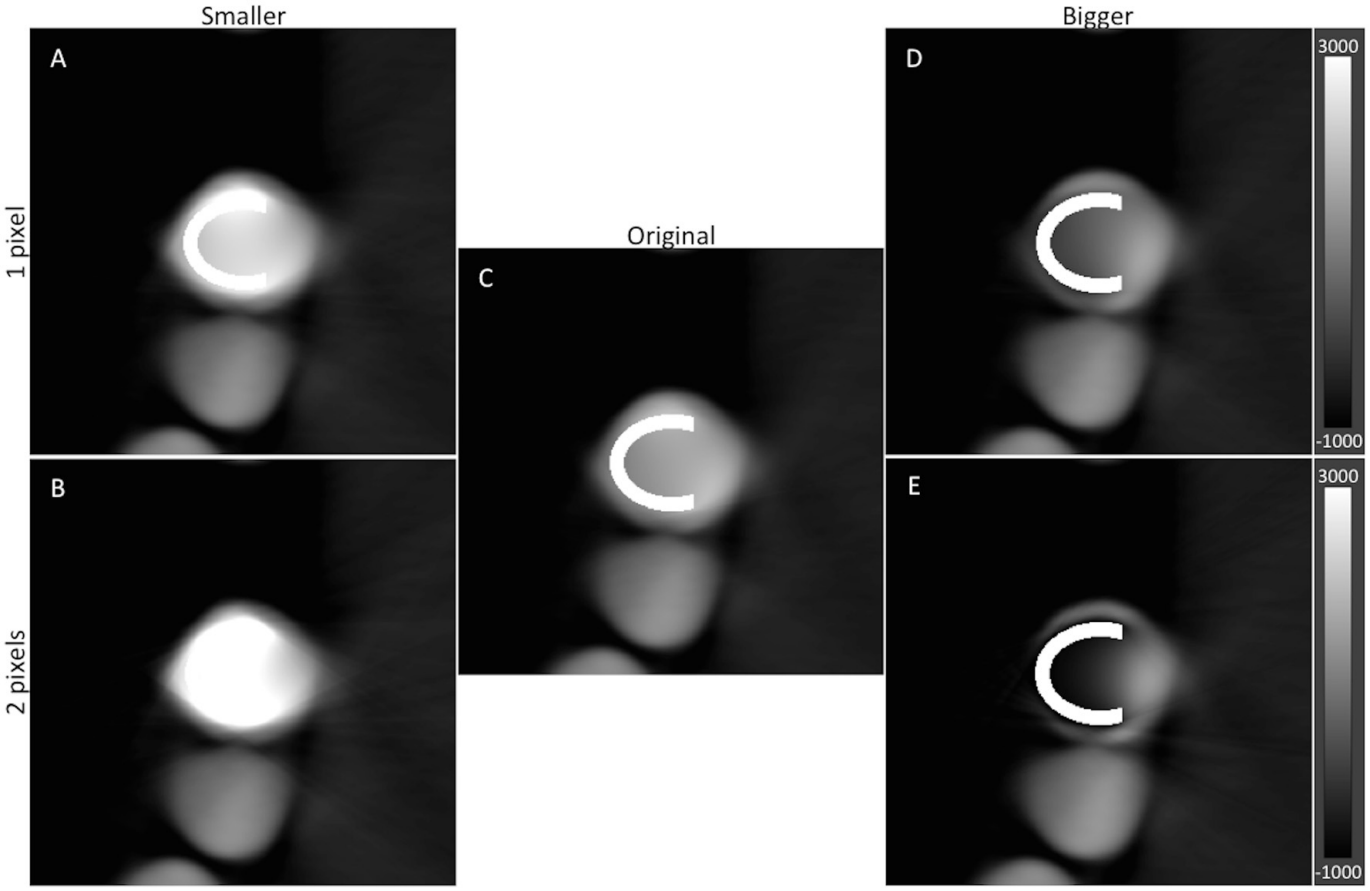
The influence of the prior information on the outcome of the model-based iterative reconstruction. The results with a smaller (A and B) or larger (D and E) metal implant show that inaccurate prior results in a less than optimal image. The reconstruction result with the original model is shown in C for comparison. All images have WW of 4000 HU and WL of 1000 HU.

Further, we investigate the influence of accurate localization and detection of the metal implant on the performance of our SPIR algorithm. Fig 8 shows the result of reconstruction presuming that the prior information on the metal implant is inaccurate; i.e. the metal implant is smaller or larger than the original size by 1 to 2 pixels. From the images, one can notice that SPIR suffers badly under such conditions. Artificially induced artifacts in the form of bright or dark shadings appear near the metal implant, resulting in the loss of details near the implant.

## Discussions

In this work, we propose an algorithm that combines spectral information and statistical reconstruction to reduce metal artifacts caused by the presence of high Z-materials. We demonstrate that the projection data generated from photon-counting detectors (PCD) can be accurately decomposed into several basis functions, providing additional knowledge on the components in the underlying scanned object. This information can be used as a prior into a penalized maximum log-likelihood iterative reconstruction, in which the true spatial location and density are enforced and corrected. We tested our algorithm with Monte Carlo simulated data of jaw phantoms that contain various shapes of dental implants. The results from our algorithm are promising, where a significant reduction of streaks in the image, elimination of bright and dark shadings, and the preservation of edges and anatomical details especially near the metal implant can be seen.

Previous work has shown the advantages of model-based reconstruction [5], in which prior information is incorporated into a reconstruction process to reduce artifacts as a result of the presence of high-Z number materials. The KCR method yields a significant reduction of streaks as well as the dark shades near the metal, while preserving the anatomical information in the background. Nevertheless, CAD based reconstruction is very dependent on the exact information of the component in the image; an inaccurate prior may result in a less than optimal image as confirmed by our findings in Fig 8. This problem will be challenging in daily clinical routine as the metal components are sometimes deformed due to prostheses experiencing significant wear prior to imaging. Our SPIR algorithm can overcome this difficulty by performing material decomposition on the projection data and consequently the location and shape of the metal component can be detected with minor discrepancies, as evident by the images in Fig 5. Further, the ability to accurately detect any shape of implant indicates that our method can be generalized and extended to other part of the body such as extremity or spine.

Another feature of our algorithm is the ability to generate pseudo-monochromatic projection data from the decomposed sinogram. This is particularly advantageous as this minimizes errors during reconstruction, thus producing images of high quality. In CT the attenuation of photons is material and energy dependent as shown in (1). Lower energy photons are more rapidly absorbed than higher energy photons. However, in most CT reconstruction algorithm the x-ray energy is averaged; thus the energy-dependency is neglected, causing the occurrence of beam hardening artifacts [1, 4, 23]. This error can be mitigated in multi energy CT or Spectral CT due to the more accurate modeling of the energy and material dependence of the x-ray attenuation, which enables the calculation of a pseudo-monochromatic projection data at different energy levels.

We note that the successfulness of the material decomposition technique is limited to the information of chemical composition of the metal implant. In this work, we used metal implant made of pure gold that has a distinctive k-edge property. Implants made out of a mixture of several unknown metals (alloys) may pose a challenge to the decomposition technique. However, this technique works well as long as the chemical composition of the metal is known beforehand so that accurate basis functions are chosen for decomposition.

In a conventional detector, incident photons are converted to optical photons at the scintillator and these signals are further amplified by the photomultiplier. These analog signals are then integrated and converted to digital signals. The whole detection and amplification processes are inefficient, while the digitization may induce some noise in the signal sampling. On the other hand, photon-counting detectors count single photons, thus it is already discrete in nature. The concept of electron holes avoids amplification and conversion process, making photon-counting detectors efficient and ‘noise-free’. This has several advantages. Firstly, due to the ‘absence’ of electronic noise at the detector, low dose acquisition protocol is possible without compromising the quality of the CT image. Secondly, low energy photons that contain valuable contrast information can be correctly detected. In an energy-integrating detector, low-energy photons may get ‘mixed’ with electronic noise. However, in energy resolving single photon-counting detector such problem does not arise, thus valuable information contained in the low-energy photons can be preserved.

The additional information provided by SCT is valuable for a variety of clinical applications—for example quantitative K-edge imaging [18], the usage of high-Z contrast agents [33, 34] and plaque detection and characterization [34, 35]. Nevertheless, photon-counting spectral CT scanners are still unavailable in the in the clinics due to several technical limitations. The slow read-out rate restricts the ability of PCD to detect high x-ray flux, resulting to pulse pile-up and photons wrongly discriminated at the detector. Currently, photon-counting detectors are able to measure photon flux levels up to 50Mcps/mm^2^ [19, 35]. In comparison, photon flux up to 10^8^ photons s^-1^ mm^-2^ is common in conventional CT. In addition, pulse splitting due to K-fluorescence from Cd (26.7 keV) or Te (37.8 keV) atoms of the detector elements will also contribute to inaccurate photon counting and discrimination. Many techniques and methods are in development to overcome these technical limitations [19]. If these technical hurdles can be overcome and such detectors can be deployed clinically, one could foresee the integration of spectral information to improve the diagnostic image quality while possibly reducing the radiation dose to the general patient population.

In this paper, we focus on the possibility of dental implant artifact reduction by incorporating spectral information obtained from PCDs. We have demonstrated the ability of our algorithm in detecting metal component in projection space. However, the same technique can also be used in detecting other k-edge containing material such as contrast agent, thus we see the potential SCT playing an integral part in clinical diagnostics. In conclusion, we have demonstrated that the information provided by SCT will be a central key in medical imaging, especially in overcoming image quality issues in clinical CT such as metal artifacts.

## References

[1] Barrett JF, Keat N. Artifacts in CT: recognition and avoidance. Radiographics. 2004;24(6):1679–91. Epub 2004/11/13. 10.1148/rg.246045065 . 15537976

[2] Hsieh J. Computed Tomography Principles, Design, Artifacts, and Recent Advances: Wiley; 2009.

[3] Brooks RA, Di Chiro G. Beam hardening in x-ray reconstructive tomography. Physics in medicine and biology. 1976;21(3):390 778862 10.1088/0031-9155/21/3/004

[4] De Man B, Nuyts J, Dupont P, Marchal G, Suetens P. Metal streak artifacts in X-ray computed tomography: a simulation study Nuclear Science Symposium, 1998 Conference Record 1998 IEEE: IEEE; 1998 p. 1860–5.

[5] Stayman JW, Otake Y, Prince JL, Khanna AJ, Siewerdsen JH. Model-based tomographic reconstruction of objects containing known components. IEEE Trans Med Imaging. 2012;31(10):1837–48. Epub 2012/05/23. 10.1109/TMI.2012.2199763 . 22614574 PMC4503263

[6] De Man B, Nuyts J, Dupont P, Marchal G, Suetens P. Reduction of metal streak artifacts in x-ray computed tomography using a transmission maximum a posteriori algorithm. IEEE transactions on nuclear science. 2000;47(3):977–81

[7] Wang G, Snyder DL, O'Sullivan JA, Vannier MW. Iterative deblurring for CT metal artifact reduction. IEEE Trans Med Imaging. 1996;15(5):657–64. Epub 1996/01/01. 10.1109/42.538943 . 18215947

[8] Robertson DD, Yuan J, Wang G, Vannier MW. Total hip prosthesis metal-artifact suppression using iterative deblurring reconstruction. Journal of Computer Assisted Tomography. 1997;21(2):293–8. 10.1097/00004728-199703000-00024 WOS:A1997WM65500023. 9071303

[9] Koehler T, Brendel B, Brown KM. A New Method for Metal Artifact Reduction in CT The International Conference in X-ray Computed Tomography; Salt Lake City 2011.

[10] Watzke O, Kalender WA. A pragmatic approach to metal artifact reduction in CT: merging of metal artifact reduced images. European radiology. 2004;14(5):849–56. Epub 2004/03/12. 10.1007/s00330-004-2263-y . 15014974

[11] Glover GH, Pelc NJ. An algorithm for the reduction of metal clip artifacts in CT reconstructions. Med Phys. 1981;8(6):799–807. Epub 1981/11/01. . 7322078 10.1118/1.595032

[12] Kalender WA, Hebel R, Ebersberger J. Reduction of CT artifacts caused by metallic implants. Radiology. 1987;164(2):576–7. Epub 1987/08/01. 10.1148/radiology.164.2.3602406 . 3602406

[13] Meyer E, Raupach R, Lell M, Schmidt B, Kachelriess M. Normalized metal artifact reduction (NMAR) in computed tomography. Med Phys. 2010;37(10):5482–93. Epub 2010/11/26. . 21089784 10.1118/1.3484090

[14] Meyer E, Raupach R, Lell M, Schmidt B, Kachelriess M. Frequency split metal artifact reduction (FSMAR) in computed tomography. Med Phys. 2012;39(4):1904–16. Epub 2012/04/10. 10.1118/1.3691902 . 22482612

[15] Fischer P, Helmich A, Lindner M, Wermes N, Blanquart L. A photon counting pixel chip with energy windowing. Ieee Transactions on Nuclear Science. 2000;47(3):881–4. 10.1109/23.856711 WOS:000088378300031.

[16] Shikhaliev PM. Computed tomography with energy-resolved detection: a feasibility study. Phys Med Biol. 2008;53(5):1475–95. Epub 2008/02/26. 10.1088/0031-9155/53/5/020 . 18296774

[17] Shikhaliev PM. Energy-resolved computed tomography: first experimental results. Phys Med Biol. 2008;53(20):5595–613. Epub 2008/09/19. 10.1088/0031-9155/53/20/002 . 18799830

[18] Roessl E, Proksa R. K-edge imaging in x-ray computed tomography using multi-bin photon counting detectors. Phys Med Biol. 2007;52(15):4679–96. Epub 2007/07/20. 10.1088/0031-9155/52/15/020 . 17634657

[19] Schlomka JP, Roessl E, Dorscheid R, Dill S, Martens G, Istel T, et al Experimental feasibility of multi-energy photon-counting K-edge imaging in pre-clinical computed tomography. Phys Med Biol. 2008;53(15):4031–47. Epub 2008/07/10. 10.1088/0031-9155/53/15/002 . 18612175

[20] Alvarez RE, Macovski A. Energy-selective reconstructions in X-ray computerized tomography. Phys Med Biol. 1976;21(5):733–44. Epub 1976/09/01. . 967922 10.1088/0031-9155/21/5/002

[21] Johnson TR, Krauss B, Sedlmair M, Grasruck M, Bruder H, Morhard D, et al Material differentiation by dual energy CT: initial experience. European radiology. 2007;17(6):1510–7. Epub 2006/12/08. 10.1007/s00330-006-0517-6 . 17151859

[22] Engel KJ, Herrmann C, Zeitler G. X-ray scattering in single- and dual-source CT. Med Phys. 2008;35(1):318–32. Epub 2008/02/26. 10.1118/1.2820901 . 18293587

[23] Fessler JA. Statistical image reconstruction methods for transmission tomography Handbook of Medical Imaging, Volume 2 Medical Image Processing and Analysis. Bellingham, WA: SPIE Press; 2000.

[24] Erdogan H, Fessler JA. Ordered subsets algorithms for transmission tomography. Physics in medicine and biology. 1999;44(11):2835 10588288 10.1088/0031-9155/44/11/311

[25] Lange K. Convergence of EM image reconstruction algorithms with Gibbs smoothing. Medical Imaging, IEEE Transactions on. 1990;9(4):439–46. 18222791 10.1109/42.61759

[26] Noeël P, Voigt J, Penchev P, Renger B, Rummeny E, Fiebich M. WE-C-110-11: A Monte-Carlo Based Software-Bench for Performance Characterization of CT Reconstruction Algorithms. Medical Physics. 2011;38(6):3811 10.1118/1.3613350

[27] Kawrakow I, Mainegra-Hing E, Tessier F, Walter B. The EGSnrc C++ class library. NRC Report PIRS-898 (rev A). 2009.

[28] Ljungberg M, Strand S, King MA. Monte Carlo Calculations in Nuclear Medicine: Applications in Diagnostic Imaging: CRC Press; 2012.

[29] Kawrakow I, Mainegra-Hing E, Rogers DWO, Tessier F, Walter BRB. The EGSnrc Code System: Monte Carlo Simulation of Electron and Photon Transport 6th printing ed Ottawa, Canada: National Research Council of Canada; 2013.

[30] Berger M, Hubbell J. XCOM, Photon cross sections on a personal computer: The Bureau; 1987.

[31] Noel PB, Walczak AM, Xu J, Corso JJ, Hoffmann KR, Schafer S. GPU-based cone beam computed tomography. Computer methods and programs in biomedicine. 2010;98(3):271–7. Epub 2009/09/29. 10.1016/j.cmpb.2009.08.006 . 19782424

[32] Fehringer A, Lasser T, Zanette I, Noël PB, Pfeiffer F, editors. A versatile tomographic forward-and back-projection approach on multi-GPUs SPIE Medical Imaging; 2014: International Society for Optics and Photonics.

[33] Roessl E, Cormode D, Brendel B, Engel KJ, Martens G, Thran A, et al Preclinical spectral computed tomography of gold nano-particles. Nuclear Instruments & Methods in Physics Research Section a-Accelerators Spectrometers Detectors and Associated Equipment. 2011;648:S259–S64. 10.1016/J.Nima.2010.11.072 WOS:000305376900064.

[34] Cormode DP, Roessl E, Thran A, Skajaa T, Gordon RE, Schlomka JP, et al Atherosclerotic plaque composition: analysis with multicolor CT and targeted gold nanoparticles. Radiology. 2010;256(3):774–82. Epub 2010/07/30. 10.1148/radiol.10092473 20668118 PMC2923725

[35] Feuerlein S, Roessl E, Proksa R, Martens G, Klass O, Jeltsch M, et al Multienergy photon-counting K-edge imaging: potential for improved luminal depiction in vascular imaging. Radiology. 2008;249(3):1010–6. Epub 2008/10/14. 10.1148/radiol.2492080560 . 18849505

