# Correction: Reduction of Metal Artifact in Single Photon-Counting Computed Tomography by Spectral-Driven Iterative Reconstruction Technique

**DOI:** 10.1371/journal.pone.0131500

**Published:** 2015-06-22

**Authors:** Radin A. Nasirudin, Kai Mei, Petar Penchev, Andreas Fehringer, Franz Pfeiffer, Ernst J. Rummeny, Martin Fiebich, Peter B. Noël

The third author’s name is spelled incorrectly. The correct name is: Petar Penchev. The correct citation is: Nasirudin RA, Mei K, Penchev P, Fehringer A, Pfeiffer F, Rummeny EJ, et al. (2015) Reduction of Metal Artifact in Single Photon-Counting Computed Tomography by Spectral-Driven Iterative Reconstruction Technique. PLoS ONE 10(5): e0124831. doi:10.1371/journal.pone.0124831
